# Elucidation of the molecular mechanisms underlying adverse reactions associated with a kinase inhibitor using systems toxicology

**DOI:** 10.1038/npjsba.2015.5

**Published:** 2015-09-28

**Authors:** Takahiro Amemiya, Masashi Honma, Yoshiaki Kariya, Samik Ghosh, Hiroaki Kitano, Yoshihisa Kurachi, Ken-ichi Fujita, Yasutsuna Sasaki, Yukio Homma, Darrel R Abernethy, Haruki Kume, Hiroshi Suzuki

**Affiliations:** 1 Department of Pharmacy, The University of Tokyo Hospital, Faculty of Medicine, The University of Tokyo, Tokyo, Japan; 2 Laboratory of Pharmacology and Pharmacokinetics, The University of Tokyo Hospital, Faculty of Medicine, The University of Tokyo, Tokyo, Japan; 3 The Systems Biology Institute, Tokyo, Japan; 4 Integrated Open Systems Unit, Okinawa Institute of Science and Technology, Okinawa, Japan; 5 Sony Computer Science Laboratories, Inc., Tokyo, Japan; 6 Laboratory for Disease Systems Modeling, RIKEN Center for Integrative Medical Sciences, Kanagawa, Japan; 7 Department of Pharmacology, Graduate School of Medicine, Osaka University, Osaka, Japan; 8 Institute of Molecular Oncology, Showa University, Tokyo, Japan; 9 Division of Medical Oncology, Department of Medicine, Showa University School of Medicine, Tokyo, Japan; 10 Department of Urology, The University of Tokyo Hospital, Faculty of Medicine, The University of Tokyo, Tokyo, Japan; 11 Office of Clinical Pharmacology, Office of Translational Sciences, US Food and Drug Administration, Silver Spring, MD, USA

## Abstract

**Background/Objectives:**

Targeted kinase inhibitors are an important class of agents in anticancer therapeutics, but their limited tolerability hampers their clinical performance. Identification of the molecular mechanisms underlying the development of adverse reactions will be helpful in establishing a rational method for the management of clinically adverse reactions. Here, we selected sunitinib as a model and demonstrated that the molecular mechanisms underlying the adverse reactions associated with kinase inhibitors can efficiently be identified using a systems toxicological approach.

**Methods:**

First, toxicological target candidates were short-listed by comparing the human kinase occupancy profiles of sunitinib and sorafenib, and the molecular mechanisms underlying adverse reactions were predicted by sequential simulations using publicly available mathematical models. Next, to evaluate the probability of these predictions, a clinical observation study was conducted in six patients treated with sunitinib. Finally, mouse experiments were performed for detailed confirmation of the hypothesized molecular mechanisms and to evaluate the efficacy of a proposed countermeasure against adverse reactions to sunitinib.

**Results:**

*In silico* simulations indicated the possibility that sunitinib-mediated off-target inhibition of phosphorylase kinase leads to the generation of oxidative stress in various tissues. Clinical observations of patients and mouse experiments confirmed the validity of this prediction. The simulation further suggested that concomitant use of an antioxidant may prevent sunitinib-mediated adverse reactions, which was confirmed in mouse experiments.

**Conclusions:**

A systems toxicological approach successfully predicted the molecular mechanisms underlying clinically adverse reactions associated with sunitinib and was used to plan a rational method for the management of these adverse reactions.

## Introduction

Targeted kinase inhibitors are an important class of therapeutics that are widely used to treat various types of cancer,^1,2^ and a large number of new compounds are under clinical development.^3^ Although these compounds show improved toxicity profiles compared with classical anticancer chemotherapeutics, the tolerability of kinase inhibitors is often limited, which can hamper their performance with respect to improving clinical outcomes.^4,5^ Although kinase inhibitors are designed and selected to inhibit specific kinases at the drug discovery stage, subsequent studies and clinical experience have revealed that kinase inhibitors also interact with various off-target molecules, i.e., those not intended as pharmacological targets, with off-target interactions leading to unanticipated adverse reactions.^6,7^ To maximize the clinical benefits of kinase inhibitors, it is desirable to develop a rational approach to improve their tolerability. Despite the development of novel technologies that enable comprehensive measurements of off-target interaction profiles,^8^ standard methods for elucidating the molecular mechanisms underlying development of adverse reactions in a clinical setting based on off-target data have not yet been established. Development of a method to identify means to mitigate adverse reactions based on the identification of toxicological targets and the molecular mechanisms is urgently required. In this study, we selected sunitinib as a model compound to demonstrate an efficient method to elucidate the molecular mechanisms underlying clinically adverse reactions associated with kinase inhibitors and to design a possible countermeasure for adverse effects.

Sunitinib is a multi-kinase inhibitor with antitumor and anti-angiogenic activities.^9^ It is indicated for the treatment of metastatic renal cell carcinoma (RCC), imatinib-resistant gastrointestinal stromal tumors, and metastatic pancreatic neuroendocrine tumors.^10^ Sunitinib targets multiple tyrosine kinases, including vascular endothelial growth factor receptors, platelet-derived growth factor receptors and stem cell factor receptors.^11^ However, despite its potent antitumor effects, sunitinib also causes numerous adverse reactions. These reactions are markedly more prevalent and severe in patients treated with sunitinib than in those treated with sorafenib, another multi-kinase inhibitor used in the treatment of RCC, even though their primary target profiles are similar.^12^ Among the adverse reactions associated with sunitinib, thrombocytopenia has an exceptionally high incidence and is generally recognized as a dose-limiting toxicity.^5^ Hyperthyrotropinemia is also more prevalent in patients treated with sunitinib than in those treated with sorafenib.^13^ Other associated adverse reactions include hepatic and cardiac dysfunction.^13^ In general, although sunitinib is currently recognized as the initial drug of choice for the treatment of advanced RCC, its toxicity is a matter of clinical concern.^14^

Understanding possible molecular mechanisms of such adverse reactions requires a systems toxicology approach where diverse data and knowledge are integrated to uncover dynamics of molecular interactions networks linked to physiological responses. It can be a power approach especially where the polypharmacological properties of a compound obscure the physiological mechanism of drug action.^15,16^ In addition, *in silico* simulation enables the examination of working hypotheses without time-consuming and costly experiments, thereby helping to develop plausible hypotheses that warrant experimental investigation. Although such approaches may ultimately contribute to the elucidation of the molecular mechanisms underlying physiological drug activities, few reports demonstrating concrete methodologies are available. In the present study, we used systems toxicology approach to efficiently elucidate the molecular mechanisms underlying the clinically adverse reactions associated with sunitinib and to identify a method to attenuate these adverse reactions based on an understanding of the molecular mechanisms.

## Materials and Methods

### Calculation of occupancy rates

The occupancy rates of sunitinib and sorafenib at various human kinases were calculated from a previous report that included the *K*_d_ values of a number of kinase inhibitors obtained with high-throughput measurements.^8^ The full list of *K*_d_ values of sunitinib and sorafenib to human kinases is available as the Supplementary Information of this article.^8^ The steady state mean unbound plasma concentrations of these agents under clinical conditions (*C*_p,u,ss_) were obtained from the information displayed on the Japanese package inserts. From a pharmacokinetic standpoint, the free drug concentration in cells is in equilibrium with the plasma-unbound concentration of the drug. Thus, the occupancy rates can be calculated using the *K*_d_ value for drug binding to the kinase and the mean plasma unbound concentration (*C*_p,u,ss_) of the drug at steady state as $\frac{C_{p,u,\text{ss}}}{K_{d}+C_{p,u,\text{ss}}}$.

### Construction of a glycogen metabolism and related pathways map

Metabolic pathway maps were constructed utilizing previous reports^17,18^ and public databases, such as PANTHER^19^ and BioModels.^20^ Specifically, the glycogen metabolic pathway was retrieved from previous reports,^17,18^ and the glycolysis pathway, tricarboxylic acid cycle and pentose-phosphate pathway were retrieved from the PANTHER database (accession numbers P00024, P00051 and P02762, respectively). The glutathione metabolic pathway was retrieved from previous reports^21,22^ and from the BioModels database (ID: BIOMD0000000268). These pathways were then integrated to create a system-wide map using CellDesigner4.3.^19,20,23^

### Model simulations

Although there are no publicly available simulation models that include the entire relationship between PHKG1/2 and GSH, models for glycogen metabolism and glycolysis,^24^ the pentose–phosphate pathway,^25^ and the glutathione metabolism pathway^26^ are available. For glycogen metabolism and the glycolysis pathway, the inhibitory effects of sunitinib were added to the model by assuming competitive inhibition of phosphorylase kinase (PHK) with an inhibition constant of 5.5 μM, a reported value for the dissociation constant.^8^ This simulation model was published as a MATLAB script in the original paper.^24^ MATLAB 2012b was used to simulate the effects of varied sunitinib concentrations on PHK content, glycogen phosphorylase and glycogen synthase activity, and glycogen and G6P content, as measured at the end of the simulation. The kinetic behavior of the pentose–phosphate pathway was described by the kinetic laws published in a previous report^25^ and the SABIO-RK database.^27^ These kinetic data were implemented into a model formed using systems biology markup language (File S2). Simulations based on this model were performed using MATLAB 2012b. The glutathione metabolism model was taken from BioModels ID: BIOMD0000000268 in systems biology markup language format, and simulations were performed on COPASI.^28^ To simulate the effect of antioxidants, a node representing vitamin E was added to the glutathione model, which assumed direct quenching of H_2_O_2_ by vitamin E.

### Cell culture

293FT cells were cultured in Dulbecco’s modified Eagle’s medium (DMEM, Nacalai Tesque, Kyoto, Japan) supplemented with 10% fetal bovine serum (FBS, Biowest, Nuaillé, France), 2 mM L-glutamine (Nacalai Tesque), and 1% penicillin-streptomycin (PCSM, Life Technologies, Carlsbad, CA, USA). HEK 293 cells were cultured in DMEM supplemented with 10% FBS and 1% PCSM. Primary hepatocytes were isolated as described previously,^29^ seeded onto collagen-coated plates, and cultured in William E medium (Sigma-Aldrich, Carlsbad, CA, USA) supplemented with 10% FBS, 2 mM L-glutamine, and 1% PCSM.

### Preparation of recombinant PHKG1/2 kinase domains

Genes encoding human and mouse PHKG1 and PHKG2 were engineered to include a His-tag at the N-terminus and then subcloned into pcDNA3.3 vectors (Life Technologies), which were then transfected into 293FT cells using Lipofectamine 2000 (Life Technologies). At 48 h post-transfection, cells were lysed in lysis buffer containing phosphate-buffered saline (pH 8.0), 1% Nonidet P-40, and a protease inhibitor cocktail (Roche, Indianapolis, IN, USA). Recombinant proteins were purified using Profinity IMAC Ni-Charged Resin (Bio-Rad, Tokyo, Japan).

### *In vitro* kinase assay

To measure the kinase activity of PHKG1/2, 100 ng of the recombinant protein was incubated with 10 μg of κ-casein (Wako, Osaka, Japan), 2 μM ATP in kinase buffer (20 mM HEPES, 10 mM MgCl_2_, 3 mM MnCl_2_ and 0.1 mg/ml bovine serum albumin, pH 7.6) and 10 μM β-mercaptoethanol (Sigma-Aldrich) for 30 min at 37 °C. The concentration of the remaining ATP was determined using a Kinase-Glo Plus luminescent kinase assay (Promega, Madison, WI, USA). To determine the concentration for 50% inhibition (IC50), drugs were added to the reaction mixture at the indicated concentrations. ATP concentrations were fitted to the standard model, and IC50 values were determined according to Powell’s nonlinear least squares method with a uniform weighting factor. Analyses were performed using Scientist software (Micromath, St Louis, MO, USA).

### Clinical observations

Patients with advanced RCC scheduled for sunitinib treatment were recruited for the observational study. Written informed consent was obtained from each patient and blood samples were collected at the indicated times.

### Animal models and drug administration

Six-week-old male C57BL6/J mice were purchased from Japan SLC (Shizuoka, Japan) and fed an MF diet (Oriental Yeast, Tokyo, Japan) containing sunitinib (0.167 mg/g MF diet; estimated as 26.7 mg/kg/day) or sorafenib (0.129 mg/g MF diet; estimated as 20.6 mg/kg/day) for 14 days. The doses were adjusted to sustain plasma drug concentrations at levels comparable to those achieved in patients. To evaluate the effects of the concomitant use of an antioxidant, mice were fed a diet containing both α-tocopherol nicotinate (0.189 mg/g MF diet; estimated as 30 mg/kg/day) and sunitinib for 14 days. To examine the contribution of oxidative stress on sunitinib hepatotoxicity, buthionine sulfoximine was administered intraperitoneally (6 mmol/kg at −1 and 11 h) and sunitinib was administered orally (8 mg/kg at 0 h and 6 mg/kg at 6, 12 and 18 h). After 36 h, the mice were killed, and their organs and cells of interest were analyzed.

### Measurement of clinical test values and metabolites

Serum ALT levels were measured using an L-type Wako GPT J2 (Wako) kit and a Dimension Xpand analyzer (Siemens, Berlin, Germany) according to the manufacturers’ protocols. To measure tissue glycogen and G6P levels and the NADPH/NADP+ ratio, excised tissue samples were homogenized with the extraction buffers provided in the glycogen assay kit (BioVision, San Francisco, CA, USA), the glucose-6-phosphate assay kit (BioVision), or the NADP+/NADPH quantification kit (BioVision), and then centrifuged at 20,000*g* for 5 min at 4 °C. Supernatants were analyzed according to the manufacturers’ protocols. Tissue glutathione levels were measured in excised tissue samples homogenized with 6% metaphosphoric acid (Wako) and centrifuged at 20,000*g* for 5 min at 4 °C. Supernatants were divided into two aliquots, one of which was used to assay total glutathione, while 2-vinylpyridine (Nacalai Tesque) was added to the other sample to mask GSH. Total glutathione and GSSG were assayed at an absorbance of 405 nm in the presence of 25% triethanolamine (Nacalai Tesque), 6 mM 5,5’-dithiobis-(2-nitrobenzoic acid) (Nacalai Tesque), 0.3 mM NADPH (Wako), and 5 units/ml of glutathione reductase (Sigma-Aldrich). Troponin T measurements were conducted by SRL (Tokyo, Japan) using an electrochemiluminescence immunoassay. The N-terminal fragment of the prohormone B-type natriuretic peptide (NT-proBNP) was measured using an NT-proBNP ELISA kit (USCN Life Science, Wuhan, China) according to the manufacturer’s protocol. Platelet counts were performed by Mitsubishi Chemical Medience Corporation (Tokyo, Japan) using an electrical resistivity method. Thyroid-stimulating hormone levels were measured using a rodent thyroid-stimulating hormone assay kit (Endocrine Technologies, Newark, CA, USA) according to the manufacturer’s protocol. Total triiodothyronine (T3), total thyroxin (T4), free T3 (FT3) and free T4 (FT4) concentrations were measured using an ARCHITECT i1000SR analyzer (Abbott Japan, Katsuyama, Japan) with the corresponding kits.

### Quantification of drug concentrations

Plasma drug concentrations were measured using liquid chromatography coupled with tandem mass spectrometry (ACQUITY UPLC-Quattro Premier XE system, Waters, Milford, MA, USA) in a device equipped with an ACQUITY UPLC BEH shield (RP18, 1.7 μm) and a 2.1×100 mm column (Waters). All the data were acquired and processed using MassLynx software (version 4.1) with QuanLynx (Waters). Positive-ion electrospray tandem mass spectroscopy, operated under the multiple reaction monitoring mode, was used to detect mass transitions (parent to daughter ion), with *m/z* 399.48 to 283.1 for sunitinib, *m/z* 371.27 to 283.1 for *N*-desethylsunitinib, *m/z* 465.35 to 252.1 for sorafenib, and *m/z* 268.23 to 116.03 for metoprolol, which served as an internal standard. Plasma samples were deproteinized with 4 volumes of acetonitrile (Nacalai Tesque) and the supernatants were used for analysis.

### *In vivo* knockdown experiments

A small hairpin RNA (shRNA) sequence that inhibits murine PHKG2, specifically, 5′-GCCTTAAGCAGTCACCGTTTA-3′, was designed using BLOCK-iT RNAi Designer (Invitrogen, Carlsbad, CA, USA). The shRNA duplex contains a loop sequence (5´-TTCAAGAGA-3′) that was initially subcloned into an RNAi Ready pSIREN vector (Clontech Laboratories, Mountain View, CA, USA) and then into pAdeno-X (Clontech Laboratories), which was digested with *Pac*I and transfected into HEK 293 cells to yield adenoviruses encoding shRNAs. Both virus production and amplification were performed according to the manufacturer’s protocols. A CsCl density gradient ultracentrifugation was used to purify crude adenoviral preparations. The viral titer was determined using an Adeno-X Rapid Titer Kit (Clontech Laboratories). Mice were intravenously injected with 1×10^9^ infectious units of adenovirus. Seventy-two hours after adenovirus administration, the mice were killed, and the relevant tissues and cells were analyzed.

### Quantification of mRNA expression by real-time PCR

Total RNA was extracted from mouse liver using RNA isoplus reagent (TaKaRa, Shiga, Japan). Reverse transcription was performed using ReverTra Ace (Toyobo Engineering, Osaka, Japan) and mRNA expression levels were measured using real-time quantitative PCR with SYBR GreenER qPCR SuperMix Universal (Life Technologies), an Eco Real-Time PCR System (Illumina, San Diego, CA, USA) and the provided software. The following primers were used: 5′-GCACCAGAGATCCTTAAAT-3′ and 5′-TAGCATCAGGATTTGGCGC-3′ for mouse PHKG2, and 5′-CCGGAAGGAAAACTGACAGC-3′ and 5′-GTGGTGGTGAAGCTGTAGCC-3′ for β-actin.

### Statistical analyses

All the data are expressed as the mean±s.d. of at least five independent experiments. Statistical analysis was performed using Student’s *t*-test, analysis of variance followed by the Tukey–Kramer *post hoc* test, or analysis of covariance, where applicable.

### Study approval

Experiments using human samples were conducted according to a study protocol approved by the Institutional Review Board of the Graduate School of Medicine, The University of Tokyo. All animal procedures were approved by the Institutional Animal Care and Use Committee of the Graduate School of Medicine, The University of Tokyo.

## Results

### *In silico* simulations indicate that off-target inhibition of phosphorylase kinase gamma 1/2 by sunitinib decreases cellular glutathione levels

**Figure 1:**
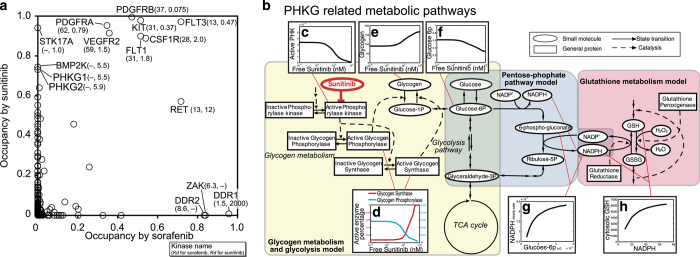
*In silico* simulations indicate that off-target inhibition of phosphorylase kinase gamma 1/2 by sunitinib decreases cellular glutathione levels. (**a**) Comparison of the kinase occupancy profiles of sunitinib and sorafenib. Kinase occupancy was estimated from the mean plasma unbound concentration and *K*_d_ value for each drug. (**b**) Schematic diagram depicting metabolic pathways of glycogen metabolism created from previous reports and knowledge-based information. Phosphorylase kinase (PHK) activates glycogen phosphorylase, an enzyme involved in glycogen catabolism. Glycogen metabolism is linked to glycolysis via glucose-6-phosphate (G6P), which is also shared by the pentose–phosphate pathway. The pentose–phosphate pathway is adjacent to the system regulating glutathione redox balance via production of nicotinamide adenine dinucleotide phosphate (NADPH). (**c**–**f**) The effect of sunitinib on the metabolites of glycogen metabolism and glycolysis are simulated. Sunitinib inhibits PHK activity (**c**) and glycogen phosphorylase activity (**d**), and increases glycogen synthase activity (**d**), leading to glycogen accumulation (**e**). Finally, sunitinib administration decreases G6P levels (**f**). (**g**) The effect of the sunitinib-induced lower levels of G6P on NADPH levels in the pentose–phosphate pathway is simulated. This simulation indicates that lower levels of G6P decrease NADPH levels. (**h**) The effect of lower levels of NADPH (induced by sunitinib) on glutathione (GSH) levels in the glutathione metabolism pathway. This simulation indicates that lower NADPH levels lead to lower GSH levels.

Candidate kinases potentially responsible for the adverse reactions associated with sunitinib were identified by comparing two compounds that share primary target kinases, sunitinib and sorafenib, on the basis of the assumption that differences in the prevalence and severity of the adverse reactions induced by these drugs are related to preferential inhibition of off-target kinases by sunitinib. Kinase occupancy profiles for the two drugs were calculated from their mean unbound plasma concentrations^30,31^ and reported dissociation constant (*K*_d_) values^8^ (Figure 1a). A comparison of the two profiles identified four candidate kinases: serine/threonine kinase 17a (STK17A), phosphorylase kinase gamma 1 (PHKG1), PHKG2 and BMP2 inducible kinase (BMP2K). Only a limited number of reports are available on the physiological roles of STK17A and BMP2K. STK17A was first identified as a proapoptotic human kinase,^32^ and rodents were shown to lack a homologous gene.^33^ Some reports indicated that STK17A induction in cancer cells enhances cell sensitivity to anticancer agents.^34^ BMP2K was first isolated from prechondroblastic cells treated with BMP-2 and was shown to negatively regulate osteoblast differentiation.^35^ On the basis of these data, it seems unlikely that development of the prevalent adverse reactions associated with sunitinib can be explained by STK17A or BMP2K inhibition. In contrast, PHKG1 and PHKG2 were reported to act as catalytic subunits for distinct isoforms of phosphorylase kinase, a kinase that regulates the activation of glycogen phosphorylase, a rate-limiting enzyme in glycogen catabolism.^17,36^ Phosphorylase kinase is ubiquitously expressed,^37^ although the distribution of the catalytic subunit, either PHKG1 or PHKG2, is tissue-specific, with the former expressed in skeletal muscle, heart and thyroid, and the latter in liver and testis.^36^ Sunitinib inhibits both isoforms, thereby disrupting glucose metabolism, which is essential for the normal physiological function of all tissues, particularly those associated with adverse reactions to sunitinib. Thus, the influence of sunitinib-mediated PHKG1/2 inhibition on physiological homeostasis was selected for investigation. First, we used *in vitro* kinase assays to confirm that sunitinib inhibited the kinase activity of human and mouse PHKG1/2 at levels comparable with those calculated from reported *K*_d_ values (Supplementary Figure S1). Next, a metabolic map was created by integrating information on glycogen metabolism and associated regulatory systems from public databases^19,20^ and previous reports^17^ (Supplementary Figure S2). This comprehensive map indicated that glycogen metabolism is linked to glycolysis via glucose-6-phosphate (G6P),^17^ which is also located at the gateway to the pentose–phosphate pathway (Figure 1b). The pentose–phosphate pathway is the only pathway in mammalian cells that produces the reduced form of nicotinamide adenine dinucleotide phosphate (NADPH),^38^ which regulates the glutathione redox balance. The reduced form of glutathione (GSH) is a typical radical scavenger involved in eliminating the reactive oxygen species produced by numerous physiological processes.^39^ However, it remains unclear whether GSH redox balance is affected by phosphorylase kinase inhibition or the influence of phosphorylase kinase inhibition is diminished before affecting GSH redox balance owing to the buffering or feedback effects of metabolic pathways. Therefore, *in silico* simulations were used to examine whether sunitinib-mediated inhibition of PHKG1/2 affects GSH levels. Although models of glycogen metabolism and glycolysis,^24^ the pentose–phosphate pathway,^25^ and glutathione metabolism^26^ are available, to the best of our knowledge, there is currently no single available model that includes the entire relationship between PHKG1/2 and GSH. Therefore, we performed sequential simulations using all these models (Supplementary Figure S3). To simulate the effects of sunitinib, sunitinib-mediated inhibition of PHKG1/2 was added to the model of glycogen metabolism.^24^ The activities of related kinases or the levels of various metabolites were plotted against the sunitinib concentration (Figures 1c–f). The simulation indicated that sunitinib induced glycogen accumulation and G6P reduction by altering the activity of the relevant enzymes. Next, we simulated the effect of reduced G6P levels on the pentose–phosphate pathway.^25^ The results indicated that a decrease in the initial content of G6P decreased the ratio of NADPH to NADP^+^ (the oxidized form of NADPH; Figure 1g). Finally, we performed simulations of the glutathione metabolic pathway^26^ and found that the lower NADPH content decreased GSH levels (Figure 1h). Collectively, the sequential simulations suggest that sunitinib-mediated inhibition of PHKG1/2 lowers GSH levels, likely causing oxidative stress in various organs and tissues.

### Clinical observations confirm that glycogen accumulation and oxidative stress are associated with sunitinib administration

**Figure 2:**
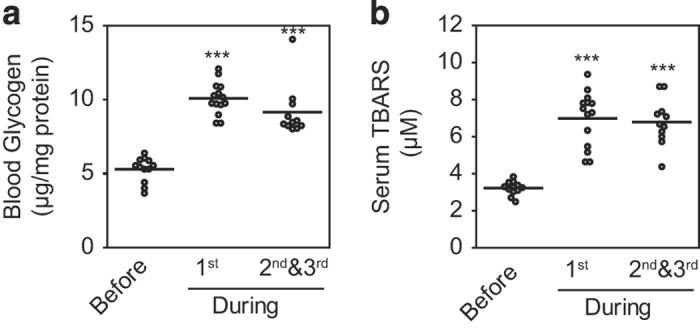
Sunitinib induces glycogen accumulation and oxidative stress in patients with renal cell carcinoma. (**a** and **b**) Six patients with advanced renal cell carcinoma (RCC) who were scheduled for treatment with sunitinib were enrolled in the study. Blood samples were collected before sunitinib treatment, weekly during the first course of treatment, and every 2 weeks during the second and third courses. Sunitinib administration increased blood glycogen content (**a**) and serum thiobarbituric acid reactive substance (TBARS) levels (**b**). Each circle represents a measured value, and each horizontal line represents the mean of the measured values. ***P<0.001 versus before sunitinib treatment.

A clinical observational study was conducted to validate the above *in silico* simulation results. To determine whether glycogen accumulation and oxidative stress occur in patients treated with sunitinib (as predicted by the *in silico* simulations), six patients receiving sunitinib as a therapy for advanced or relapsing RCC were enrolled in the study (Supplementary Table S1). Blood samples were collected before sunitinib treatment, once per week during the first course of treatment, and once every 2 weeks during the second and third courses. Changes in blood glycogen and serum thiobarbituric acid reactive substance (TBARS) levels, which reflect lipid peroxide levels, were analyzed. Sunitinib therapy increased both blood glycogen (Figure 2a) and serum TBARS levels (Figure 2b) in all the six patients, indicating the development of oxidative stress. These results demonstrate that sunitinib induces glycogen accumulation and oxidative stress in clinical patients and suggest that the *in silico* simulations successfully predicted the unfavorable drug actions associated with sunitinib-mediated off-target kinase inhibition.

### Experimental confirmation that sunitinib-mediated inhibition of PHKG2 induces oxidative stress in the liver

**Figure 3:**
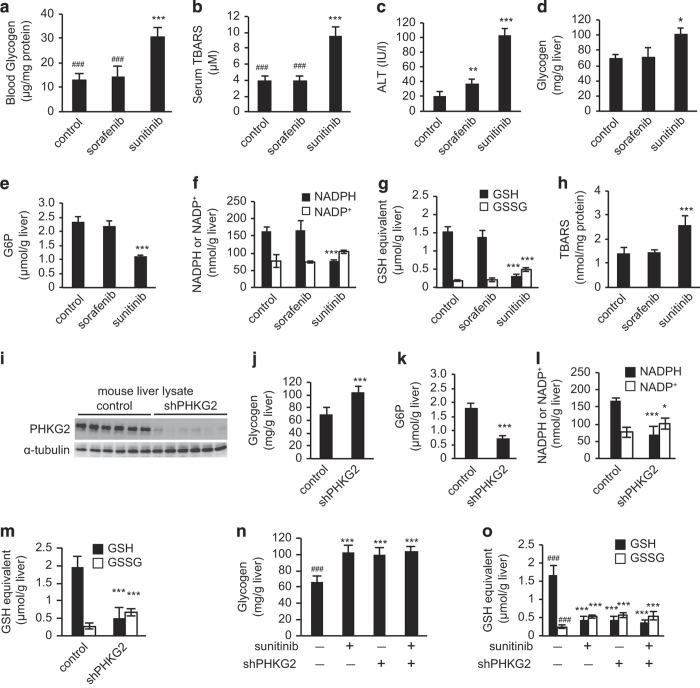
*In vivo* experiments confirm *in silico* simulation predictions that oxidative stress is induced in the liver by inhibiting phosphorylase kinase gamma 2 (PHKG2). (**a**–**h**) Mice were fed a diet containing sunitinib or sorafenib for 14 days, after which serum and blood biomarkers and hepatic metabolite levels were analyzed. Blood glycogen content (**a**) and serum thiobarbituric acid reactive substance (TBARS) levels (**b**) are higher in the sunitinib-treated group than those in the untreated control and sorafenib-treated groups, consistent with the clinical observations shown in Figure 2. Serum alanine aminotransferase (ALT) (**c**) is also elevated in the sunitinib-treated group, indicating the development of liver damage. Sunitinib administration increases hepatic glycogen content (**d**), decreases hepatic glucose-6-phosphate (G6P) content (**e**), decreases the ratio of nicotinamide adenine dinucleotide phosphate (NADPH) to NADP^+^ in the liver (**f**) and decreases the ratio of glutathione (GSH) to the oxidized form, GSSG, in the liver (**g**). In addition, the hepatic TBARS level (**h**), a measure of oxidative stress, is higher in the sunitinib-treated group than that in control animals or in the sorafenib-treated group. All animal data are expressed as the mean±s.d. (*n*=6). *P<0.05, **P<0.01, ***P<0.001 versus control. ^#^*P*<0.05, ^##^*P*<0.01, ^###^*P*<0.001 versus the sunitinib-treated group. (**i**–**m**) Mice were treated with a small hairpin RNA (shRNA) targeting *PHKG2* (shPHKG2) or control adenovirus, and the effects were determined 3 days later. Knockdown of hepatic PHKG2 expression (**i**) is confirmed with immunoblot analysis. The livers of mice treated with the shPHKG2 adenovirus show increased glycogen content (**j**) and decreased G6P content (**k**), together with a reduction in the NADPH/NADP^+^ ratio (**l**) and GSH/GSSG ratio (**m**). In addition, sunitinib administration does not show additive effects with respect to hepatic glycogen content (**n**) and the GSH/GSSG ratio (**o**). Data are expressed as the mean±s.d. (*n*=6). *P<0.05, **P<0.01, ***P<0.001 versus control. ^#^*P*<0.05, ^##^*P*<0.01, ^###^*P*<0.001 versus the sunitinib-treated group.

To further validate the *in silico* predictions, we used an animal model to examine the metabolite content of those tissues adversely affected by sunitinib. Six-week-old male C57BL/6 mice were treated with sunitinib or sorafenib for 14 days; the amount of sunitinib or sorafenib in the chow was adjusted to achieve plasma concentrations within the respective clinical concentration ranges of the two drugs (Supplementary Figure S4a). Sunitinib treatment increased blood glycogen and serum TBARS levels, whereas sorafenib had no significant effect on these two parameters (Figures 3a and b). We next examined the mice for signs of hepatotoxicity, which develops in response to sunitinib but not sorafenib. Both sunitinib and sorafenib increased the amount of the liver enzyme alanine aminotransferase (ALT) in the serum, although ALT levels were lower in the sorafenib-treated than in the sunitinib-treated animals (Figure 3c). In addition, sunitinib but not sorafenib increased hepatic glycogen content (Figure 3d), which is consistent with the results of the *in silico* simulation. The changes observed for other metabolites were also consistent with the simulation results. Specifically, in the livers of sunitinib-treated mice, hepatic G6P content was 50% lower (Figure 3e), the NADPH/NADP^+^ ratio was significantly lower (Figure 3f), and the ratio of GSH to the oxidized form (GSSG) was 90% lower than those in the livers of untreated control mice (Figure 3g), whereas no significant changes in these metabolites were observed in sorafenib-treated mice. In addition, the development of oxidative stress in the livers of sunitinib-treated mice was confirmed by measuring hepatic TBARS levels (Figure 3h). These results indicated that the *in silico* simulations successfully predicted the sunitinib-mediated disruption of metabolic homeostasis in the liver. To confirm that the sunitinib-induced disruption of hepatic redox balance was mediated by PHKG2 inhibition, *in vivo* gene silencing experiments were performed using an adenovirus encoding an shRNA targeting *PHKG2* (shPHKG2) because intravenously administered adenoviruses preferentially infect and accumulate in the liver.^40^ Indeed, hepatic *PHKG2* mRNA (Supplementary Figure S4b) and PHKG2 protein expression levels (Figure 3i) were significantly lower in shPHKG2-treated mice than in mice treated with a negative control shRNA adenovirus. As expected, shPHKG2-treated mice showed significantly higher levels of hepatic glycogen and significantly lower levels of hepatic G6P (Figures 3j and k), with significantly lower NADPH/NADP^+^ and GSH/GSSG ratios than control mice (Figures 3l and m). In addition, the effects of sunitinib administration and shPHKG2 treatment were not additive (Figures 3n and o). Overall, these results confirm that sunitinib-induced hepatic oxidative stress is mediated by the inhibition of PHKG2.

### Experimental validation that sunitinib induces oxidative stress in heart, platelets and thyroid

**Figure 4:**
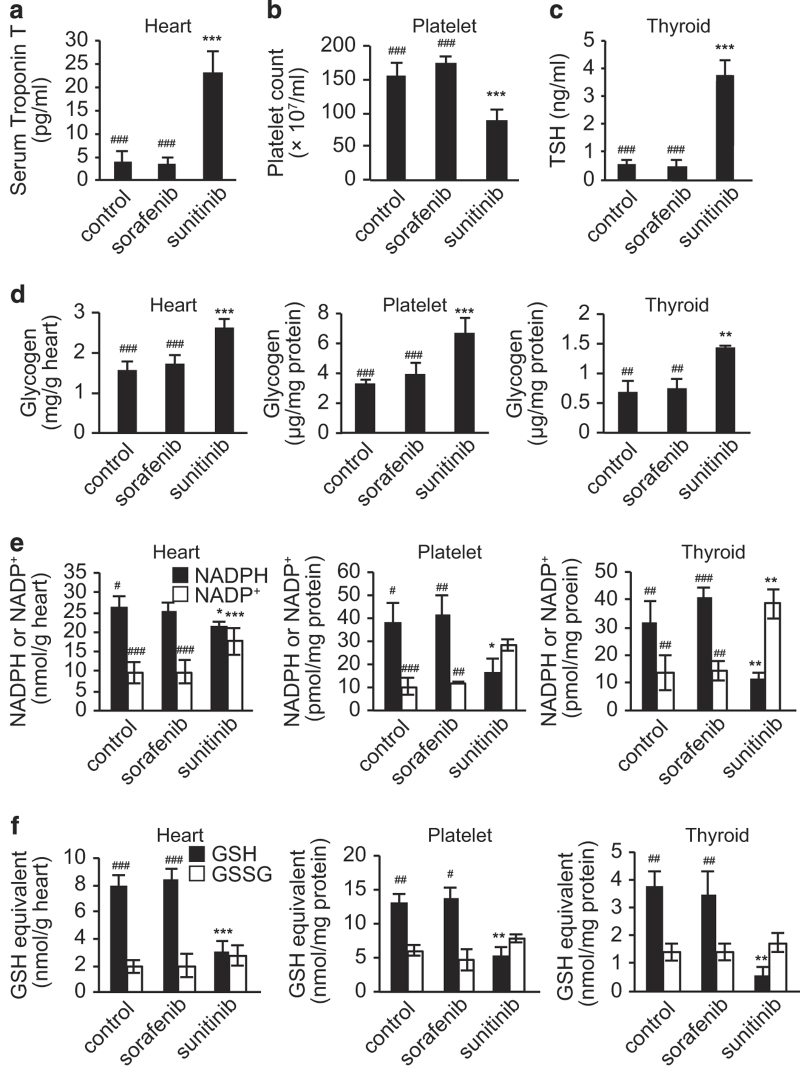
*In vivo* experimental validation shows that sunitinib induces oxidative stress in various tissues. (**a**–**f**) Mice were fed a diet containing sunitinib or sorafenib for 14 days, after which serum and blood biomarkers and metabolite levels in the heart, platelets, and thyroid glands were analyzed. Serum troponin T levels (**a**) are higher, peripheral blood platelet counts (**b**) are lower and serum thyroid-stimulating hormone (TSH) levels (**c**) are higher in the sunitinib-treated group than those in the sorafenib-treated mice, indicating the development of various adverse reactions associated with sunitinib administration. Sunitinib administration also increases glycogen content (**d**), decreases the nicotinamide adenine dinucleotide phosphate (NADPH)/NADP^+^ ratio (**e**) and decreases the ratio of glutathione (GSH) to the oxidized form, GSSG, (**f**) in the heart, platelets and thyroid gland. Data are expressed as the mean±s.d. (*n*=6). *P<0.05, **P<0.01, ***P<0.001 versus control group. ^#^ *P*<0.05, ^##^ *P*<0.01, ^###^ *P*<0.001 versus the sunitinib-treated group.

Sunitinib treatment is associated with cardiac disorders, reduced platelet counts and thyroid dysfunction.^41^ Therefore, we examined the influence of sunitinib administration on glycogen-related metabolic pathways and oxidative stress generation in the mouse heart, platelets and thyroid. Mice were administered sunitinib or sorafenib for 14 days as described above, and clinical tests were performed to confirm the development of adverse reactions. Notably, serum concentrations of troponin T, a marker indicative of cardiocyte damage, were significantly higher in the sunitinib-treated group than those in the control or sorafenib-treated groups (Figure 4a). In addition, peripheral blood platelet counts were significantly lower and serum concentrations of thyroid-stimulating hormone were significantly higher in the sunitinib group than those in the control or sorafenib groups (Figures 4b and c). Next, the metabolite content of these tissues was measured. Similar to the results shown above for the liver, sunitinib increased the glycogen content and decreased the NADPH/NADP^+^ and GSH/GSSG ratios in the heart, thyroid and platelets compared with control and sorafenib groups (Figures 4d–f). In the sunitinib-treated group, elevation of the NADP^+^ level was higher in thyroid tissue than in other tissues. This might be partly because, in thyroid tissue, NADPH is consumed to produce thyroid hormone as well as to reduce GSSG.^42^ These results again confirm that the *in silico* simulations accurately predict the sunitinib-induced disruption of metabolic homeostasis and generation of oxidative stress in these tissues and cells.

### Vitamin E alleviates adverse reactions associated with sunitinib

**Figure 5:**
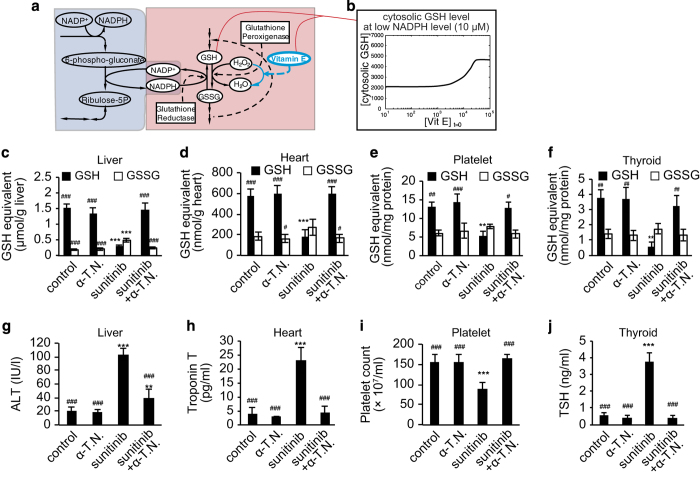
Vitamin E alleviates adverse reactions associated with sunitinib. (**a**) The effect of vitamin E, which directly traps hydrogen peroxide (H_2_O_2_), is added to the model of glutathione metabolism. (**b**) The effect of vitamin E administration on glutathione (GSH) levels is simulated. This simulation suggests that concomitant use of vitamin E potentially rescues the reduced GSH levels observed after sunitinib administration in mice. (**c**–**f**) Mice were fed a diet containing sunitinib with or without α-tocopherol nicotinate (α-TN) for 14 days, after which serum and blood biomarkers and tissue metabolite levels were analyzed. Concomitant use of α-TN with sunitinib prevents sunitinib-induced decreases in the GSH/GSSG ratio in the liver (**c**), heart (**d**), platelets (**e**) and thyroid gland (**f**). Concomitant use of α-TN with sunitinib also ameliorates the adverse reactions associated with sunitinib administration, including changes in serum alanine aminotransferase (ALT) levels (**g**), serum troponin T levels (**h**), peripheral blood platelet count (**i**) and serum thyroid-stimulating hormone (TSH) levels (**j**). Data are expressed as the mean±s.d. (*n*=6). *P<0.05, **P<0.01, ***P<0.001 versus the control. ^#^*P*<0.05, ^##^*P*<0.01, ^###^*P*<0.001 versus the sunitinib-treated group.

As a result of these simulations and experiments, we formulated a hypothesis: if oxidative stress mediates the various adverse reactions associated with sunitinib, then concomitant use of antioxidants, such as vitamin E, could alleviate those adverse effects. To verify this hypothesis, a series of *in silico* studies and animal experiments were carried out. First, we performed a simulation of the glutathione metabolism model that included vitamin E administration; a node representing vitamin E, which assumed that vitamin E directly traps hydrogen peroxide (H_2_O_2_), was added to the original model (Figure 5a). GSH levels under conditions of low NADPH, which reflects the influence of the sunitinib administration, were calculated and plotted against the initial loading concentration of vitamin E. The results showed that vitamin E loading restores GSH levels (Figure 5b). Next, we validated this *in silico* prediction by examining the *in vivo* effects of a vitamin E preparation consisting of α-tocopherol nicotinate (α-TN) in sunitinib-treated mice. Mice were administered sunitinib with or without α-TN, and the metabolite content of the tissues was measured. In all four tissues examined, co-administration of α-TN prevented sunitinib-induced decreases in the GSH/GSSG ratio, which was maintained at levels similar to those observed in the tissues of control mice (Figures 5c–f). In addition, clinical test values (for serum ALT, troponin T, thyroid-stimulating hormone and platelet counts) obtained for the co-treated animals were comparable to those obtained for the control group (Figures 5g–j), suggesting that the sunitinib-mediated oxidative stress was ameliorated by antioxidant administration. We also determined the mechanisms underlying several sunitinib-induced adverse reactions (hepatotoxicity, cardiotoxicity, thrombocytopenia and thyroid dysfunction) mediated by oxidative stress (Supplementary Figures S5–S8).

### Vitamin E has no effect on the pharmacology of sunitinib

**Figure 6:**
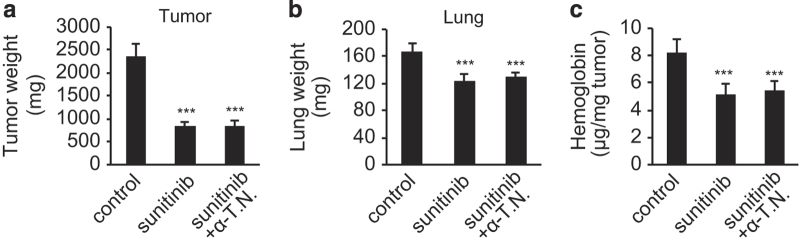
Vitamin E does not impair the anticancer effects of sunitinib. (**a**–**c**) Mice subcutaneously inoculated with Lewis lung carcinoma (LLC) cells were fed a diet containing sunitinib with or without α-tocopherol nicotinate (α-TN) for 14 days, after which the pharmacological effects of sunitinib were analyzed. The effects of sunitinib on tumor regression at the site of inoculation (**a**) and on tumor metastasis to the lungs (**b**) are not impaired by co-administration of α-TN. In addition, tumor (at the site of inoculation) hemoglobin content (**c**) is unaffected by α-TN co-administration. Data are expressed as the mean±s.d. (*n*=6). ***P<0.001 versus control.

Considering the clinical applications of sunitinib and vitamin E combination therapy, we investigated whether vitamin E negatively impacts the therapeutic efficacy of sunitinib by examining the effect of vitamin E co-administration on the anticancer efficacy of sunitinib in an animal model. Mice subcutaneously inoculated with Lewis lung carcinoma cells were administered sunitinib with or without α-TN for 14 days, after which the therapeutic effects of sunitinib on tumor growth were analyzed. Sunitinib treatment significantly reduced tumor weight (at the site of inoculation) and lung weight, which reflects the metastasis of Lewis lung carcinoma cells (Figures 6a and b). These effects were not impaired by concomitant use of α-TN (Figures 6a and b). Sunitinib primarily acts by inhibiting tumor neoangiogenesis;^43^ therefore, we also examined tumor tissue hemoglobin content, which indicates the extent of the blood supply to tumor tissues (Figure 6c). As expected, sunitinib administration decreased tumor hemoglobin content and, again, this effect was not impaired by co-administration of α-TN. These results suggest that vitamin E co-administration has little or no effect on the pharmacology of sunitinib.

## Discussion

Various targeted kinase inhibitors are available for clinical use, and these drugs have contributed to the drastic improvement in treatment outcomes for anti-cancer therapies.^1,2^ Although these agents are less toxic than classical anticancer chemotherapeutics, the spectra of adverse reactions associated with kinase inhibitors are often different from those of anticancer chemotherapies, causing an emerging problem in clinics.^4,5^ Clinical management methods for frequent adverse reactions associated with anticancer chemotherapies, such as diarrhea, nausea and myelosuppression, have been established on the basis of empirical evaluations.^44–46^ However, clinical management methods have not been well established for adverse reactions that are rarely observed by clinicians.^47^ Fast-paced launches of new kinase inhibitors might also make it more difficult to establish clinical management methods for adverse reactions in a timely manner. To overcome this problem, development of a system-oriented framework is critical so that countermeasures for adverse reactions can be designed on the basis of an understanding of the molecular mechanisms and system dynamics underlying adverse reactions. Various *in silico* tools have been developed to predict the toxicological properties of new chemical entities, including quantitative structure–activity relationship-based approaches and rule-based expert systems. However, most of these tools presupposed their utility during the drug designing steps, aiming to predict properties of a compound related to general toxicity such as genotoxicity, carcinogenicity and irritancy based on its chemical structure.^48–50^ These tools are useful for reducing the risk of discontinuation due to serious safety issues at the later stages of drug development; however, they are not intended to provide information on the detailed molecular mechanisms underlying clinically adverse reactions. Accordingly, we examined the usefulness of a systems toxicological approach in the present study and demonstrated a method to predict the reactions that may be triggered by a compound at the molecular level based on the experimental measurement of its kinase interaction profile.

The present analysis was guided by the off-target interaction profile associated with sunitinib. Off-target candidates that are affected at clinically relevant concentrations can be identified using the calculations shown in Figure 1a. It is then necessary to determine whether the effects on candidate molecules are related to the development of adverse physiological effects, i.e., to explore the associations between off-target molecules and molecules that have direct physiological effects. In cases of adverse drug reactions, the physiological responses requiring examination include broad phenomena such as apoptosis, inflammation and oxidative stress generation. Once the inputs and outputs have been determined, we can perform *in silico* simulations to examine whether a change in an input actually affects the output. The challenges in implementing this approach lie in the preparation and use of mathematical models. Obtaining quantitatively accurate predictions requires simulations using an integrated model that encompasses the entire processes of interest. However, it is not always practical to develop a large-scale integrated model and therefore, the use of multiple models that represent a subset of the entire interaction network may be necessary. A simple merger of independently derived models often results in inconsistency in the kinetic parameter values and therefore, these values have to be re-tuned after model integration. The constraint here is that fine-tuning the kinetic parameters in an integrated model may not work owing to different levels of abstraction among models. Even if the level of abstraction and methods of model description have been made consistent, it is a computationally intensive and time-consuming task that often requires additional experimental data for accurate estimates of kinetic parameter values.

**Figure 7:**
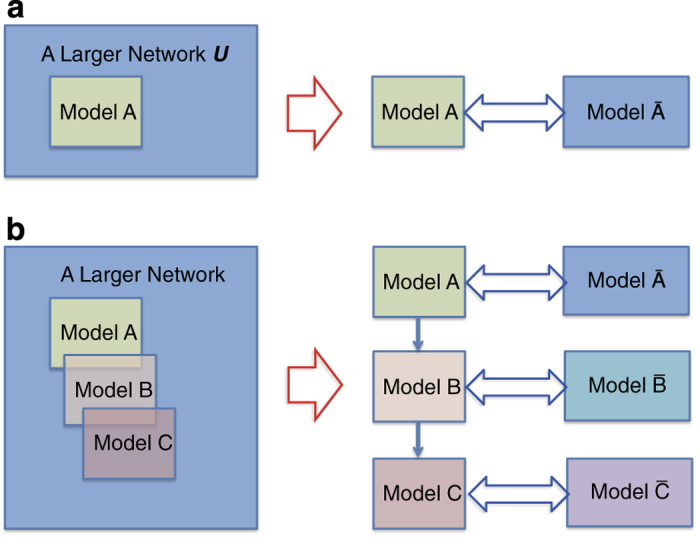
A Tandem model approach. (**a**) In general, a model represents a subset of the network (Model A) within an entire network (Model U) (Left). In this case, what is happening in practice is to implicitly consider Model A is interfacing Model $\bar{A}$ which is a black box of the rest of the network. With proper parameter tuning and value range restrictions, the model can provide reasonably accurate prediction of behaviors of the subset of the network. (**b**) Three models represent overlapping part of the network, and validated for their accuracy. This implies that these models are tuned for their counter-part black box. Within predefined range of values, a tandem application of three models is expected to deliver qualitatively useful predictions on behaviors of the network.

In this study, we applied an alternative approach that involves a tandem use of multiple models, each of which is validated to be consistent with the experiments. In general, computational models of biological networks are developed focusing on a subset of networks such as the MAPK and insulin receptor pathways and a model of the entire network is not created. Nevertheless, many of these models achieve a practically sufficient accuracy that can be used for further scientific studies and drug discovery.^51^ This degree of accuracy may be attributable to the modularity and robustness of the biological network. If the entire network is so tightly connected, perturbation of any part of the network affects the behaviors of all other parts. This results in an extremely unstable system, leading to an evolutionary dead-end.^52^ Thus, in practice, we can define a subset of the interaction network and consider the rest to be a ‘black box’ from which effects can be implicitly represented within a specific parameter setting of the model (Figure 7a). The success of this approach largely depends on appropriately defining the boundary of a focused network, so that strong interactions with a black box do not affect the dynamical behaviors of the model beyond the level adjustable by parameter settings. In this study, we used three models each representing a distinct functional pathway in the metabolic network, and they were validated experimentally (Figure 7b). Furthermore, the models were interfaced by the dose change of specific molecules. The output of the first model (glycogen metabolism and glycolysis) is a dose change of glucose and G6P that in turn became inputs for the second model (pentose–phosphate pathway). The interface between the second model and the third model (glutathione metabolism) is NADP^+^ and NADPH. Fortunately, no obvious feedback loop exists between the models that could seriously alter the behaviors of an upstream model depending on the outcome of the downstream models. It is noteworthy that the purpose of the simulation was to understand the qualitative propensity of this part of the metabolic network following sunitinib administration, rather than computing the exact dynamics with quantitative precision. The results of this tandem simulation correctly predicted physiological responses to the administration of sunitinib. Further studies may reveal conditions for this tandem approach to be practically applicable, which would lay the foundation for scaling-up computational studies efficiently for larger networks. It should be noted that we used the model to understand how sunitinib administration may qualitatively influence the metabolic system, rather than to obtain precise numerical results. To this end, the models were only required to deliver the proper degree of change in output and therefore, could have different baseline variables. In other words, the dose of the interface molecule could be 100 units in one model, and the dose level of the same molecule could be represented as 1,000 units in the other model. This is because the only information required is the degree of change and not the absolute number in each model. In such cases, a combination of simulations using separate models is expected to provide reasonable predictions.

The results reported herein demonstrate that *in silico* prediction (with appropriately designed experimental validation) is an effective strategy to examine toxicological issues associated with kinase inhibitors. Results of this study have major implications for the use of sunitinib in clinical practice, as it is essential to overcome adverse reactions associated with sunitinib tolerability.^13^ Suppression of sunitinib-mediated thyroid dysfunction may improve patients’ quality of life by ameliorating subjective symptoms, such as fatigue. More importantly, since the sunitinib-mediated reduction in platelet counts is a dose-limiting toxicity, an improvement in thrombocytopenia will allow the continued use of higher doses, which is desirable in treating the primary disease. Indeed, the therapeutic efficacy of sunitinib in RCC correlates positively with the area under the plasma concentration curve for the drug.^53^ The experiments conducted in the present study strongly indicate that the multi-organ toxicity suffered by patients receiving sunitinib may be alleviated by the concomitant administration of α-TN.

In conclusion, we demonstrated that a systems toxicological approach could be successfully utilized to elucidate molecular mechanisms underlying adverse reactions associated with kinase inhibitors. This approach may be applicable to other kinase inhibitors to maximize their clinical benefits.
