# Supplementary material for: Elucidation of the molecular mechanisms underlying adverse reactions associated with a kinase inhibitor using systems toxicology
Source: NPJ Syst Biol Appl. 2015 Sep 28;1:15005–. doi: 10.1038/npjsba.2015.5 (PMC5516806; doi:10.1038/npjsba.2015.5)
Supplement: Supplementary Information [file npjsba20155-s1.pdf]

Elucidation of the molecular mechanisms underlying adverse reactions associated with a kinase inhibitor using systems toxicology

Takahiro Amemiya, Masashi Honma, Yoshiaki Kariya, Samik Ghosh, Hiroaki Kitano, Yoshihisa Kurachi, Ken-ichi Fujita, Yasutsuna Sasaki, Yukio Homma, Darrell R. Abernethy, Haruki Kume, and Hiroshi Suzuki

Supplementary Information

Supplementary results

Sunitinib inhibits PHKG1/2 *in vitro*.

Recombinant proteins consisting of the human and mouse PHKG1/2 kinase domains fused to a His-tag at the N-terminal were obtained using a mammalian cell-expression system (Fig. S1a). *In vitro* kinase activities were then assayed in the presence or absence of sunitinib and its active metabolite *N*-desethyl sunitinib. Both compounds inhibited human and mouse PHKG1/2 kinase activity, in contrast to sorafenib, which had no effect on either enzyme (Figs. S1b–c). The *IC50* values obtained for sunitinib were comparable to previously reported K_d values for human PHKG1/2 (ref. 8).

Mathematical models and *in silico* simulation

Mathematical models of glycogen metabolism and glycolysis were created from a previously reported model with some modifications. Specifically, the inhibitory effects of sunitinib on phosphorylase kinase (PHK) activity were added to the original model (ref. 30) (Fig. S3a) by assuming competitive inhibition of PHK with an inhibition constant of 5.5 μM, which is a previously reported value for the dissociation constant between sunitinib and PHKG2 (ref. 8).

MatLab files for the models used in the present study can be found in File S1a. Simulations were performed using MatLab 2012b.

To simulate the effects of G6P on the pentose phosphate pathway, we constructed a mathematical model, which was described in SBML using CellDesigner 4.3 (File S3b), based on previously reported kinetic data for the pentose phosphate pathway (ref. 25) and data obtained from the SABIO-RK database (Fig. S3b). Simulations were performed using MatLab 2012b after extracting the ordinary differential equations from the SBML file and importing the equations into MatLab. The final time point of the simulation was set to 1,500 min, which was required for NADPH levels to reach a steady state.

NADPH effects on cellular GSH levels were simulated using a model deposited in the BioModels database (ID: BIOMD0000000268) (ref. 28). In addition, to evaluate the effects of vitamin E on GSH levels, vitamin E was added to the model by assuming direct trapping of H_2O_2 (Fig. S3c). Kinetic parameters describing the ability of vitamin E to act as a H_2O_2 scavenger were set arbitrarily and, therefore, the modeling of vitamin E is semi-quantitative because the vitamin E concentrations are arbitrary. Simulations were performed using complex pathway simulator (COPASI) as described in the original report. Simulations were performed using complex pathway simulator (COPASI) as described in the original report, and the model used is available as File S3c. For steady-state conditions, the final time point was set to 100 h.

Animal model experimental validation

To examine toxicological responses to sunitinib *in vivo*, six-week-old male C57BL/6 mice were fed sunitinib or sorafenib for 14 days, and the plasma concentration of each drug was then determined using liquid chromatography coupled with tandem mass spectrometry. The

content of sunitinib or sorafenib in mouse chow was adjusted to achieve plasma concentrations within the respective clinical concentration ranges of the two drugs (40–70 ng/mL for sunitinib and 2,500–5,000 ng/mL for sorafenib) (ref. 30, 31) (Fig. S4a).

To examine the effects of PHKG2 in the liver, *in vivo* gene-silencing experiments were performed using an adenovirus encoding an shRNA targeted against *PHKG2* (shPHKG2). Three days after adenovirus injection, total RNA was extracted from liver samples and analyzed using real-time PCR. The levels of *PHKG2* mRNA in the liver of shPHKG2-treated mice were < 30% of those in mice treated with a negative control shRNA adenovirus (shNeg) (Fig. S4b).

Detailed mechanisms of sunitinib-induced hepatotoxicity

Since oxidative stress aggravates liver injuries (ref. 54), the results shown in the main text suggested that continuous oral administration of sunitinib may increase the drug's hepatotoxicity via excess oxidative stress loading. This hypothesis was examined in mice treated intraperitoneally with buthionine sulfoximine (BSO) to deplete hepatic GSH (Fig. S5a), after which the effects of short-term administration of sunitinib were examined. Serum ALT levels were significantly higher in the sunitinib-treated mice than in the mice treated with BSO alone (Fig. S5b), supporting the hypothesis that sunitinib increases the liver's sensitivity to oxidative stress. Similarly, in a cytotoxicity assay performed in primary cultured mouse hepatocytes, BSO treatment reduced the EC_{50} of sunitinib from 136 nM to 19 nM (Fig. S5c), consistent with sunitinib-induced sensitization of oxidatively stressed hepatocytes.

Detailed mechanisms of sunitinib-induced cardiotoxicity

The heart consumes large amounts of ATP to maintain blood circulation. This is

normally supplied through β-oxidation of fatty acids and oxidative phosphorylation (ref. 55). However, a recent study in spontaneously hypertensive rats (SHRs) showed that ATP can be produced via glycolysis (ref. 56), with up-regulation of enolase and lactate dehydrogenase expression, two glycolytic enzymes, and down-regulation of enoyl-CoA hydrogenase and pyruvate dehydrogenase, enzymes involved in β-oxidation and the tricarboxylic acid cycle, respectively (ref. 56). These changes in the preferred route of energy metabolism are thought to protect cardiac tissues from excess amounts of ROS, which are generated under hypertensive conditions when ATP production proceeds via oxidative phosphorylation (ref. 56). Since hypertension is a common adverse reaction among patients who are administered VEGF signal inhibitors such as sunitinib and sorafenib, whose primary targets include VEGFRs (ref. 12), we examined energy-metabolism pathways in sunitinib- and sorafenib-treated mice. Sorafenib treatment resulted in up-regulation of enolase and lactate dehydrogenase expression and down-regulation of enoyl-CoA hydrogenase and pyruvate dehydrogenase expression (Fig. S6e), consistent with the changes observed in SHRs (ref. 56); however, sunitinib treatment had no effect on the expression of enoyl-CoA hydrogenase, pyruvate dehydrogenase, or lactate dehydrogenase, but markedly up-regulated enolase expression (Fig. S6e). There was no change in the ATP content of cardiac tissue in response to either sunitinib or sorafenib (Fig. S6f). Taken together, these results indicate that the sunitinib-mediated decrease in G6P content prevents up-regulation of glycolysis despite increased enolase expression, suggesting that ATP production depends on β-oxidation of fatty acids under sunitinib-treated conditions. Concomitantly, the low GSH/GSSG ratio in the cardiac tissue of sunitinib-treated mice prevents elimination of ROS. Both of these mechanisms likely contribute to the cardiotoxicity of sunitinib. To support this conclusion, an analysis of serum samples was performed, which examined the

relationship between sunitinib-induced oxidative stress in the heart and cardiotoxicity. The results showed that serum concentrations of troponin T (Fig. 4a) and NT-proBNP (Fig. S6g), which are serum biomarkers of cardiocyte damage and depressed cardiac output, respectively, were significantly higher in sunitinib-treated mice than in control mice or sorafenib-treated mice. Moreover, co-administration of the antioxidant α-TN completely prevented the increase in troponin T (Fig. 5h) and NT-proBNP levels (Fig. S6g), thus providing further evidence that sunitinib-induced cardiac-oxidative stress plays a major role in the development of sunitinib-mediated cardiotoxicity.

Detailed mechanisms of sunitinib-mediated thrombocytopenia

In platelets, oxidative stress triggers calcium ion influx to the cytosol, leading to up-regulation of phospholipid scramblase activity, and, in turn, presentation of phosphatidylserine (PS) to the outer surface of the plasma membrane, thereby increasing phagocytosis by macrophages and thus their elimination from circulation (ref. 15, 57, 58, 59, 60). Accordingly, platelets were collected from control and treated mice, and then binding of fluorescently labeled annexin V, which selectively binds PS, was measured. Consistent with previous reports, platelets from sunitinib-treated mice bound significantly higher amounts of annexin V than platelets from either control or sorafenib-treated mice (Fig. S7a); however, annexin V binding remained at control levels in platelets from mice concomitantly administered α-TN and sunitinib (Fig. S7a). In pulse-chase experiments in which circulating platelets were labeled *in vivo* by i.v. administration of an anti-GPIbβ rat monoclonal antibody tagged with DyLight 488, sunitinib treatment significantly reduced the elimination rate of labeled platelets (over 24 h), whereas this was not the case in control animals or in those treated with sorafenib

(Fig. S7b). Furthermore, co-administration of α-TN restored the half-life of circulating platelets to control levels (Fig. S7b), suggesting that sunitinib induces surface presentation of PS and thereby decreases circulating platelet half-life. In addition, *in vitro* experiments using RAW264.7 macrophages demonstrated increased phagocytosis of platelets collected from sunitinib-treated mice (Fig. S7c). Furthermore, platelet counts were significantly lower in sunitinib-treated mice than in control mice (Fig. 5i), and this effect could be prevented by co-administration of α-TN (Fig. 5i). Overall, these data suggest that sunitinib-mediated kinase inhibition induces oxidative stress, which, in turn, increases surface presentation of PS, thereby reducing the half-life of circulating platelets.

Detailed mechanisms of sunitinib-induced thyroid dysfunction

Thyroid hormones are produced in thyroid follicles by thyroid peroxidase (TPO) which catalyzes the iodination of thyroglobulin in a reaction requiring hydrogen peroxide (ref. 61). The latter is generated by dual oxidase 2, an enzyme expressed in follicular epithelial cells, with NADPH serving as a coenzyme (ref. 61). We therefore hypothesized that by decreasing the NADPH content of the thyroid gland, sunitinib treatment would suppress thyroid hormone production. To test this hypothesis, serum concentrations of triiodothyronine (T3) and thyroxin (T4) were measured. Free T4 (FT4) levels were slightly lower in the sunitinib-treated mice than in control or sorafenib-treated mice, whereas free T3 (FT3) levels were not significantly different (Fig. S8a). By contrast, serum thyroid-stimulating hormone (TSH) levels were much higher in sunitinib-treated mice than in control or sorafenib-treated mice (Fig. 4c). Thyroid hormone levels are known to be tightly regulated by TSH feedback (ref. 62) and the above observations are similar to symptoms that develop in patients with subclinical hypothyroidism, in

which the impaired ability of the thyroid to produce thyroid hormones results in a persistently high TSH signal to ensure sufficient levels of FT3 and FT4 (ref. 63). To examine whether a similar sequence of events occurred in response to sunitinib, sunitinib-treated mice were administered octreotide, which reduces TSH secretion from the pituitary gland, thereby abrogating the feedback effect. In these animals, serum FT3 and FT4 values were significantly below control levels, as were T3 and T4 levels in the thyroid, consistent with sunitinib-mediated suppression of thyroid hormone synthesis (Figs. S8b–c). These results indicate that subclinical hypothyroidism induced by sunitinib results from impaired glycogen metabolism and low NADPH levels, which are required for glutathione metabolism in the thyroid gland.

Supplementary References

54. Hoek JB, Pastorino JG. Ethanol, oxidative stress, and cytokine-induced liver cell injury. *Alcohol* **27**, 63-68 (2002).

55. Giordano FJ. Oxygen, oxidative stress, hypoxia, and heart failure. *The Journal of clinical investigation* **115**, 500-508 (2005).

56. Jin X, Xia L, Wang LS, Shi JZ, Zheng Y, Chen WL*, et al.* Differential protein expression in hypertrophic heart with and without hypertension in spontaneously hypertensive rats. *Proteomics* **6**, 1948-1956 (2006).

57. Mirabelli F, Salis A, Vairetti M, Bellomo G, Thor H, Orrenius S. Cytoskeletal alterations in human platelets exposed to oxidative stress are mediated by oxidative and Ca2+-dependent mechanisms. *Archives of biochemistry and biophysics* **270**, 478-488 (1989).

58. Lang KS, Lang PA, Bauer C, Duranton C, Wieder T, Huber SM*, et al.* Mechanisms of suicidal erythrocyte death. *Cellular physiology and biochemistry : international journal of experimental cellular physiology, biochemistry, and pharmacology* **15**, 195-202 (2005).

59. Sener A, Ozsavci D, Bingol-Ozakpinar O, Cevik O, Yanikkaya-Demirel G, Yardimci T. Oxidized-LDL and Fe3+/ascorbic acid-induced oxidative modifications and phosphatidylserine exposure in human platelets are reduced by melatonin. *Folia biologica* **55**, 45-52 (2009).

60. Coleman DL. Regulation of macrophage phagocytosis. *European journal of clinical microbiology* **5**, 1-5 (1986).

61. Massart C, Hoste C, Virion A, Ruf J, Dumont JE, Van Sande J. Cell biology of H2O2 generation in the thyroid: investigation of the control of dual oxidases (DUOX) activity

in intact ex vivo thyroid tissue and cell lines. *Molecular and cellular endocrinology* **343**, 32-44 (2011).

62. Panda JN, Turner CW. Thyroxine feed-back on the regulation of thyrotropin [TSH] secretion. *The Journal of physiology* **195**, 29-37 (1968).

63. Cooper DS, Biondi B. Subclinical thyroid disease. *Lancet* **379**, 1142-1154 (2012).

Table S1.

No.	Age	Sex	TNM classification	Dosage amount and dosing duration
P1	70	Female	pT4N0M1	1st course : 50mg/day for 28 days<br>2nd course : sunitinib therapy discontinued
P2	65	Female	pT1bN0M1	1st course : 50mg/day for 17 days followed by 37.5mg/day for 2 days<br>2nd course : 37.5mg/day for 28 days<br>3rd course : sunitinib therapy discontinued
P3	75	Male	pT3apN2M1	1st course : 50mg/day for 12 days<br>2nd course : 25mg/day for 20 days<br>3rd course : sunitinib therapy discontinued
P4	73	Male	pT3aN0M1	1st course : 50mg/day for 9 days<br>2nd course : 37.5mg/day for 28 days<br>3rd course : sunitinib therapy discontinued
P5	50	Male	pT3aN0M1	1st course : 50mg/day for 11 days<br>2nd course : 50mg/day for 12 days followed by 37.5mg/day for 16 days<br>3rd course : 37.5mg/day for 28 days
P6	60	Male	pT1aN0M1	1st course : 50mg/day for 23 days<br>2nd course : 50mg/day for 23 days<br>3rd course : 50mg/day for 9 days followed by 37.5mg/day for 19 days

Table S1. Characteristics of RCC patients who participated in the observation study. The patients' age, sex, TNM classification of RCC, and dosage amount and dosing duration of sunitinib are summarized.

Figure S1

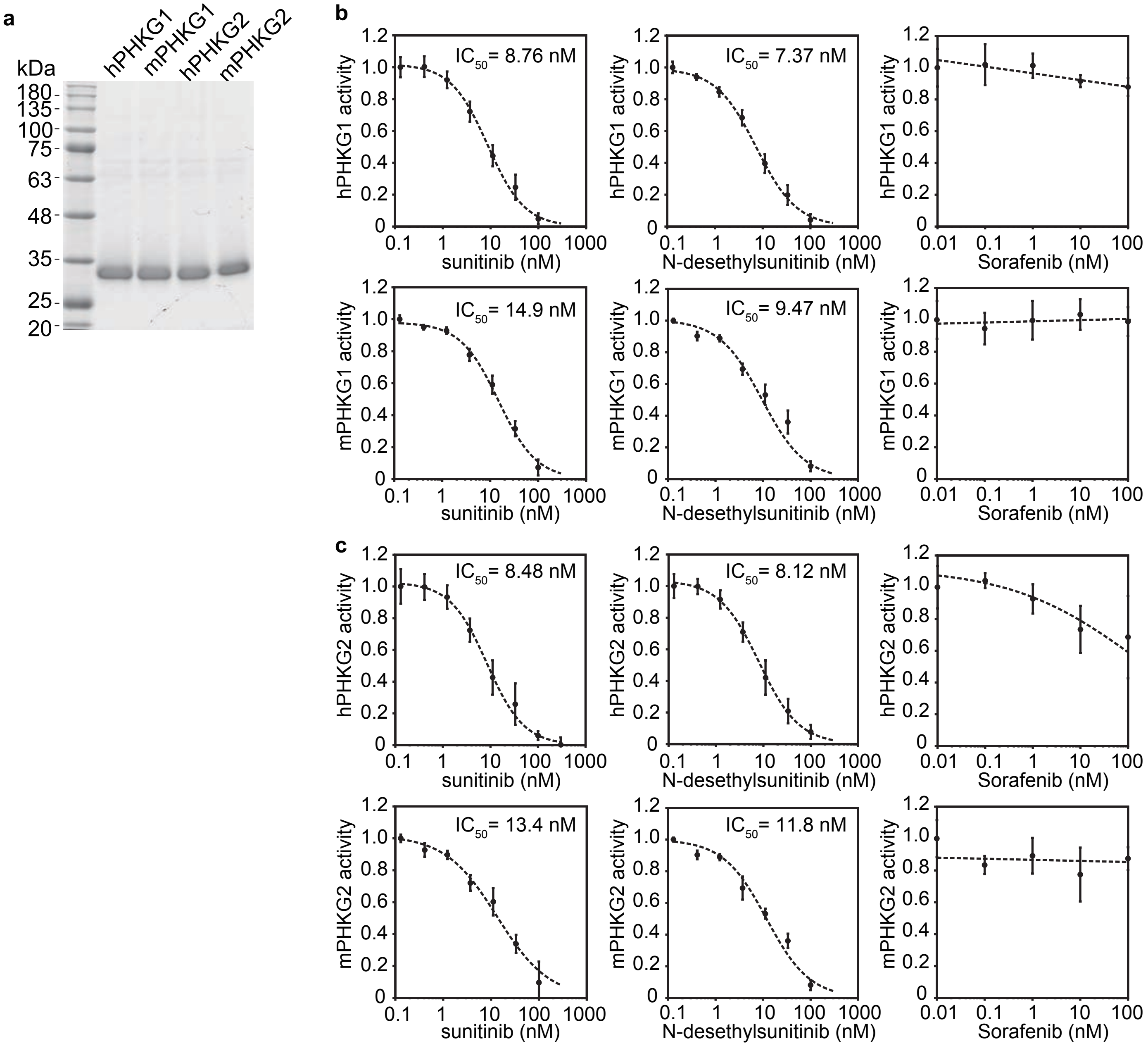

Fig. S1. PHKG1 and 2 are off-target kinases for sunitinib. **a.** SDS-PAGE analysis of recombinant proteins containing either human or mouse PHKG1/2 kinase domains, to confirm the purity of the obtained proteins. The bands were stained with Coomassie brilliant blue. **b-c .** Kinase inhibition profiles for human and mouse PHKG1 (**b**.) and for human and mouse PHKG2 (**c** .) in response to sunitinib, N-desethyl sunitinib, and sorafenib. The kinase assays were performed using -casein as a substrate, analyzing the inhibitory effect of each compound on ATP consumption. The data are expressed as the mean ± SD (n = 6).

Figure S2 PHKG related metabolic pathways

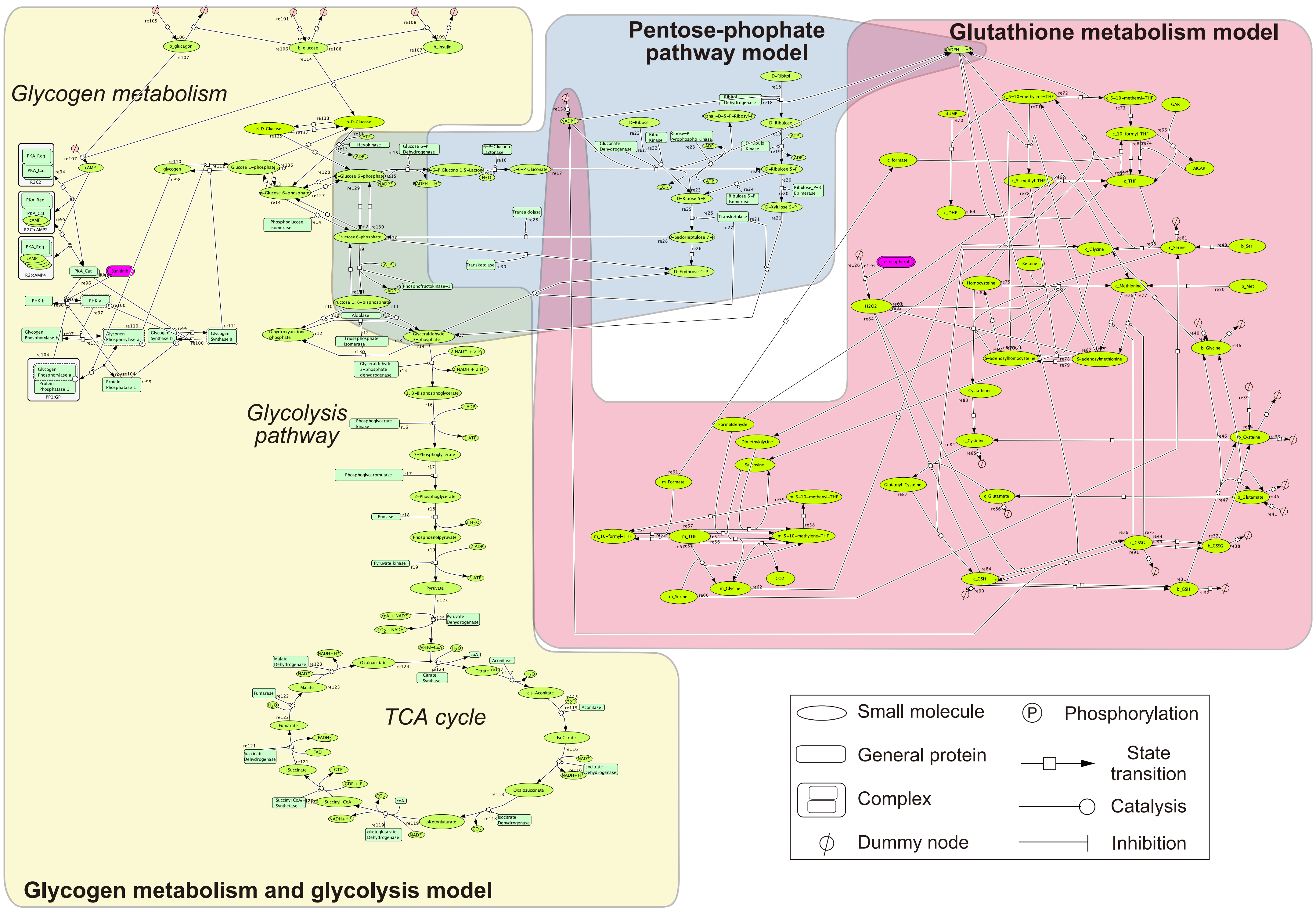

Fig. S2 PHKG related metabolic pathways A metabolic map was created by integrating information on glycogen metabolism and associated regulatory systems from public databases (ref. 19, 20) and previous reports (ref. 17). The simulation models covering these pathways were chosen to simulate the effects of sunitinib treatment.

Figure S3

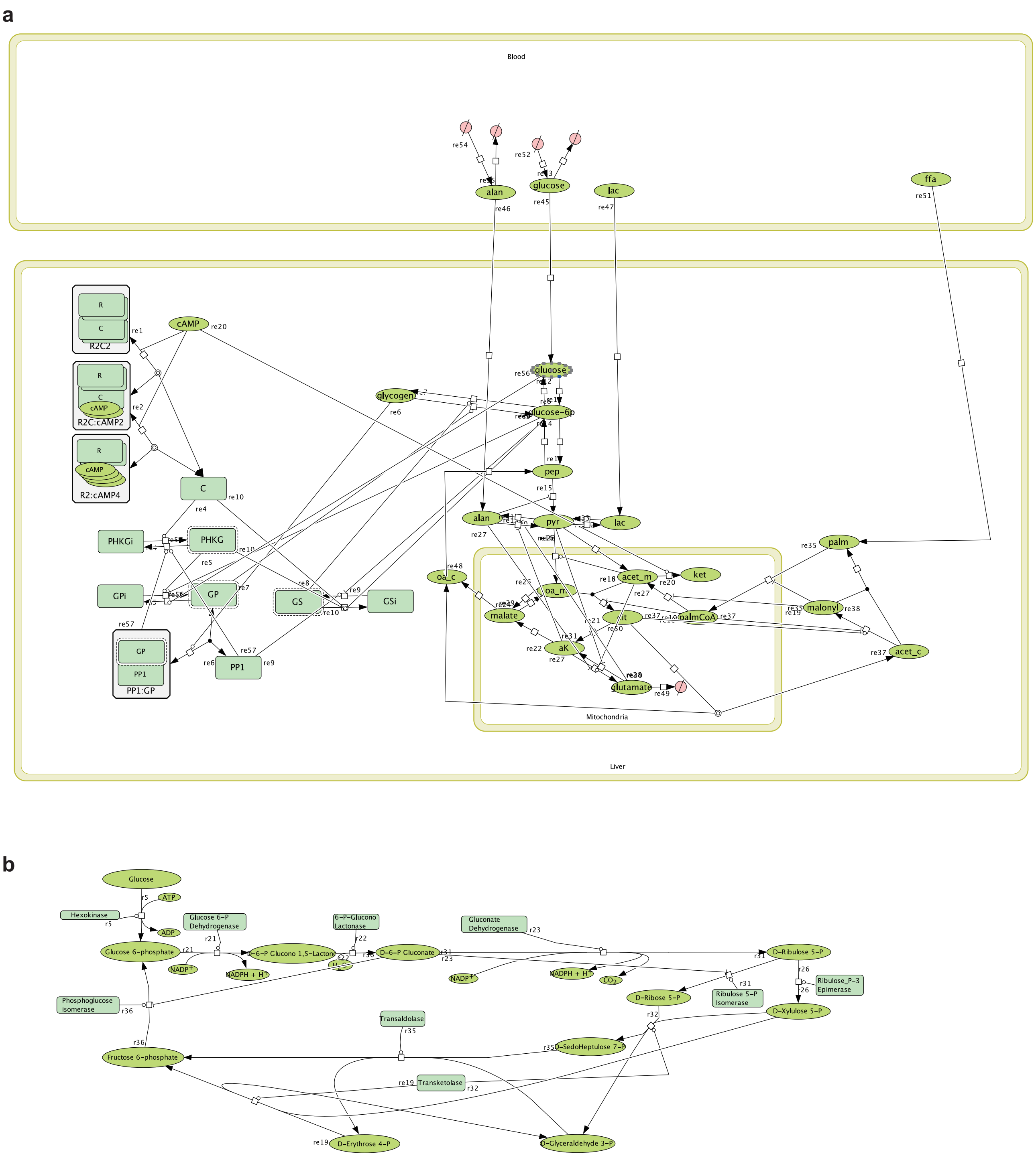

Fig. S3. Simulation models used for analyses. **a.** Glycogen and glycolysis pathway model originally built by Koenig *et al.* (ref. 24). **b.** Pentose-phosphate pathway model, whose kinetic laws and parameters were obtained from previous article (ref. 25) and from deposited parameter values in SABIO-RK.

Figure S3

c

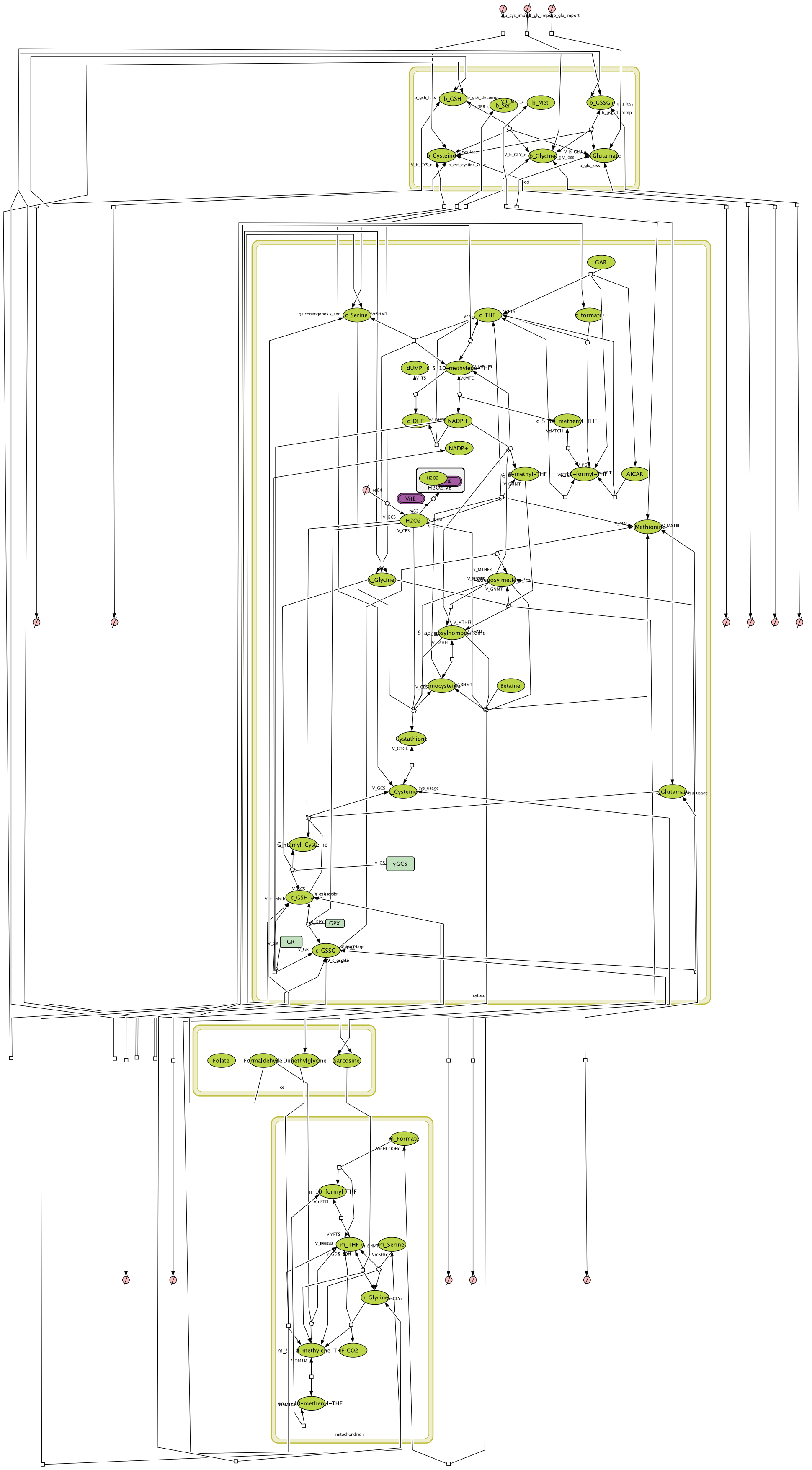

Fig. S3. (cont.) Simulation models used for analyses. c. Glutathione metabolism pathway model deposited in BioModels.net as ID: BIOMD0000000268.

Figure S4

a

	Serum concentration (ng/mL) Mean	SD	Clinical concentration (ng/mL)
Sunitinib	58.4	15.4	40 ～ 70
Sorafenib	4021	702	2500 ～ 5000

b

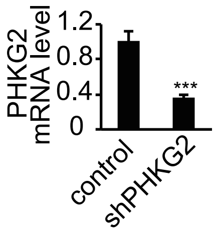

Fig. S4. Animal models used for analyses. **a.** Serum concentration of sunitinib or sorafenib in mice fed with sunitinib or sorafenib for 14 days. The both concentrations are comparable with clinical concentration ranges. **b.** Knockdown effect of shPHKG2 adenovirus on hepatic PHKG2 mRNA. *** $P < 0.001$.

Figure S5

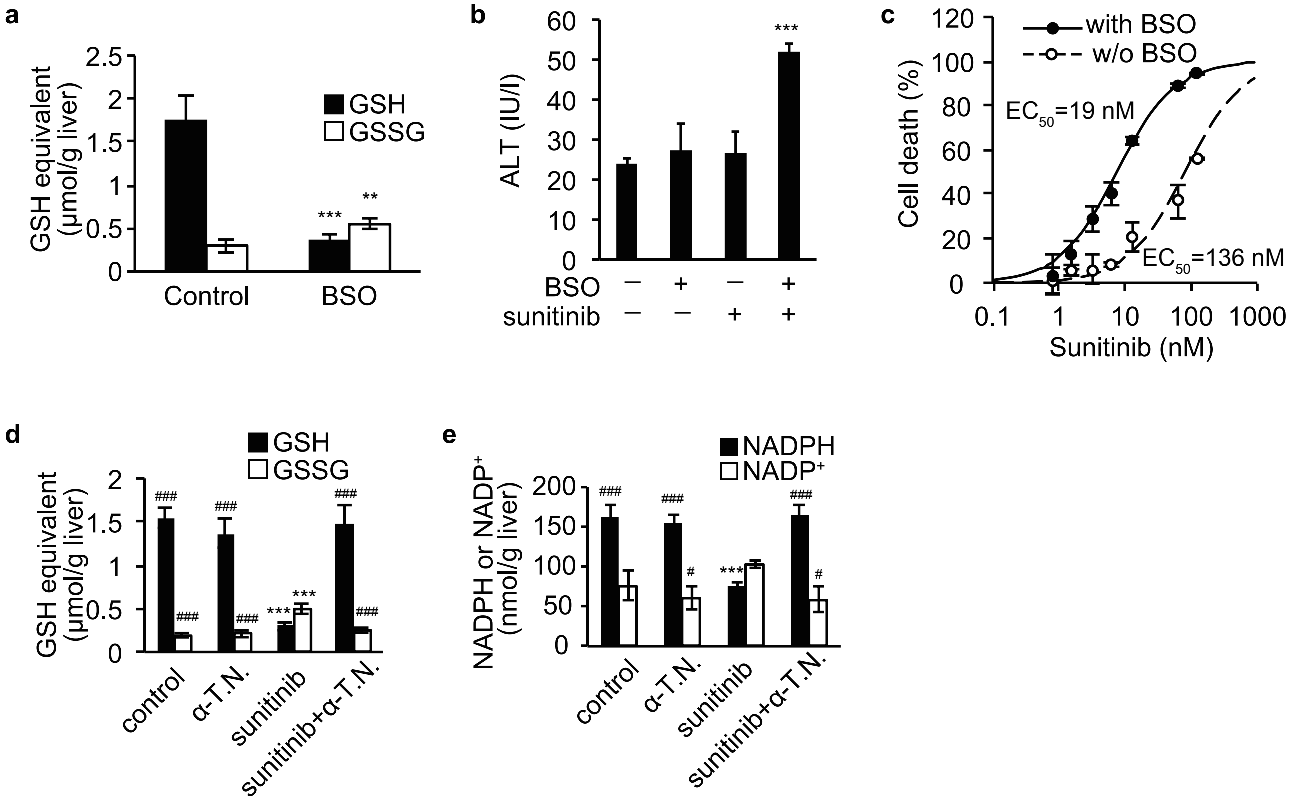

Fig. S5. Oxidative stress sensitizes hepatocytes to sunitinib toxicity. a. Confirmation of the effect of BSO treatment on the hepatic redox balance. **b.** GSH depletion enhanced the sensitivity of the liver to sunitinib toxicity following a short-term exposure to the drug *in vivo*. **c.** GSH depletion enhanced the sensitivity of hepatocytes to sunitinib toxicity *in vitro*. **d-e.** The ratios of GSH to GSSG (**d.**) and of NADPH to $NADP^+$ (**e.**) were maintained at the control levels in mice concomitantly administered sunitinib and α-tocopherol nicotinate (α-T.N.). All data are expressed as the mean ± SD (n = 6). * $P < 0.05$, ** $P < 0.01$, *** $P < 0.001$ v.s. Control; # $P < 0.05$, ## $P < 0.01$, ### $P < 0.001$ v.s. Sunitinib.

Figure S6

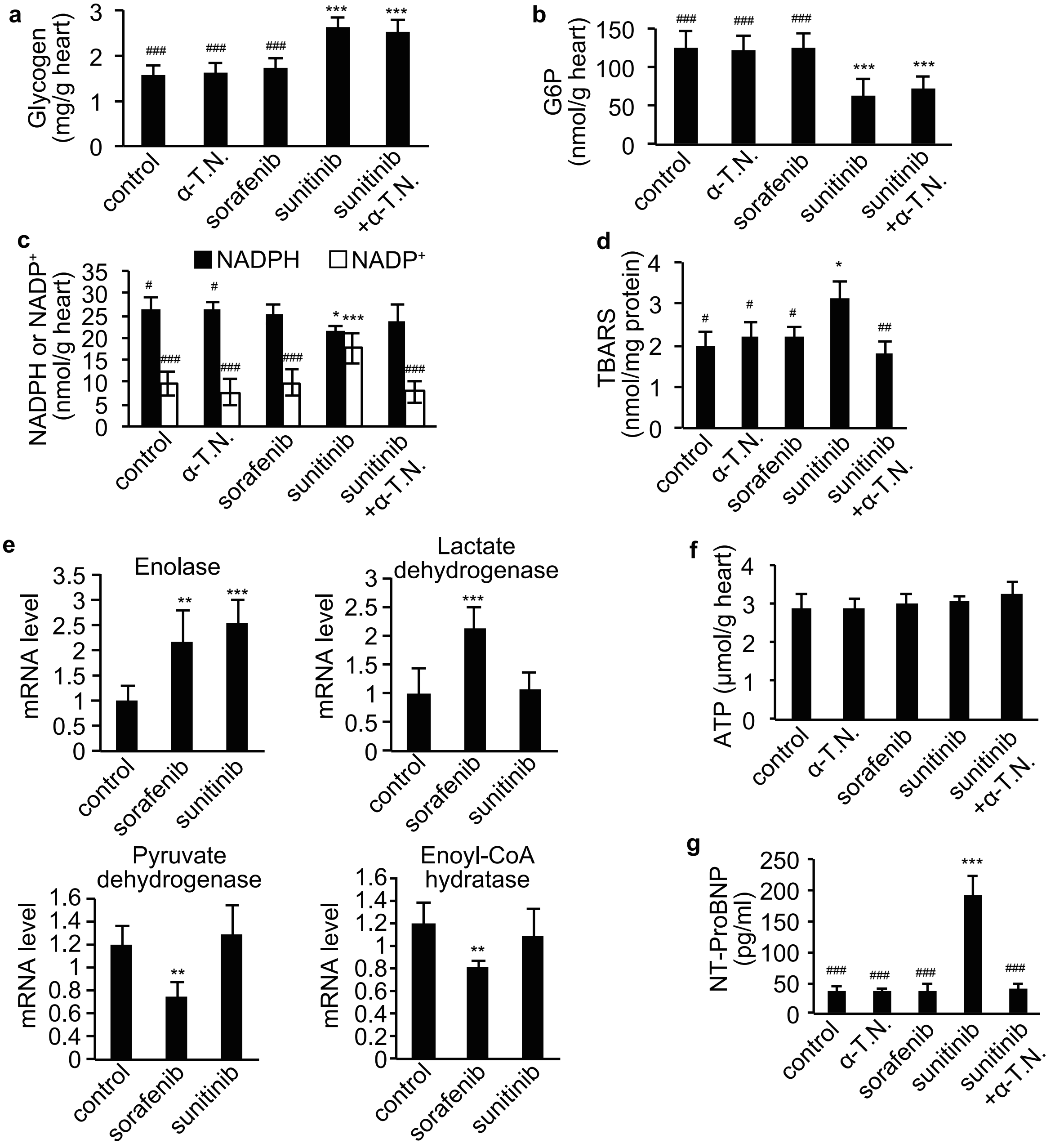

Fig. S6. Detailed mechanism of cardiac damage induced by sunitinib administration. Mice were fed sunitinib or sorafenib for 14 days after which the levels of serum biomarkers and, in the heart, glycogen metabolite concentrations and the mRNA levels of metabolic enzymes were analyzed. **a -b.** In sunitinib-treated mice, increased cardiac glycogen levels (**a**.) and decreased cardiac glucose -6-phosphate (G6P) levels (**b**.) were measured. **c.** Ratio of NADPH to $NADP^+$ in the heart was decreased in sunitinib-treated mice whereas the concomitant administration of α-tocopherol nicotinate (α-T.N.) maintained both ratios at the control level. **d.** The cardiac content of TBARS was increased in sunitinib-treated mice whereas the concomitant administration of α-T.N. resulted in the maintenance of control levels. **e -f.** mRNA expression levels of enzymes involved in energy metabolism were altered by sunitinib and sorafenib treatment (**e**.) whereas there was no change in the cardiac ATP content (**f**.). **g.** Serum concentrations of NT-proBNP, indicators of cardiac dysfunction, was increased in mice treated with sunitinib whereas the concomitant administration of α-T.N. resulted in the maintenance of control levels. All data are expressed as the mean ± SD (n = 6). * $P < 0.05$, ** $P < 0.01$, *** $P < 0.001$ v.s. Control; # $P < 0.05$, ## $P < 0.01$, ### $P < 0.001$ v.s. Sunitinib.

Figure S7

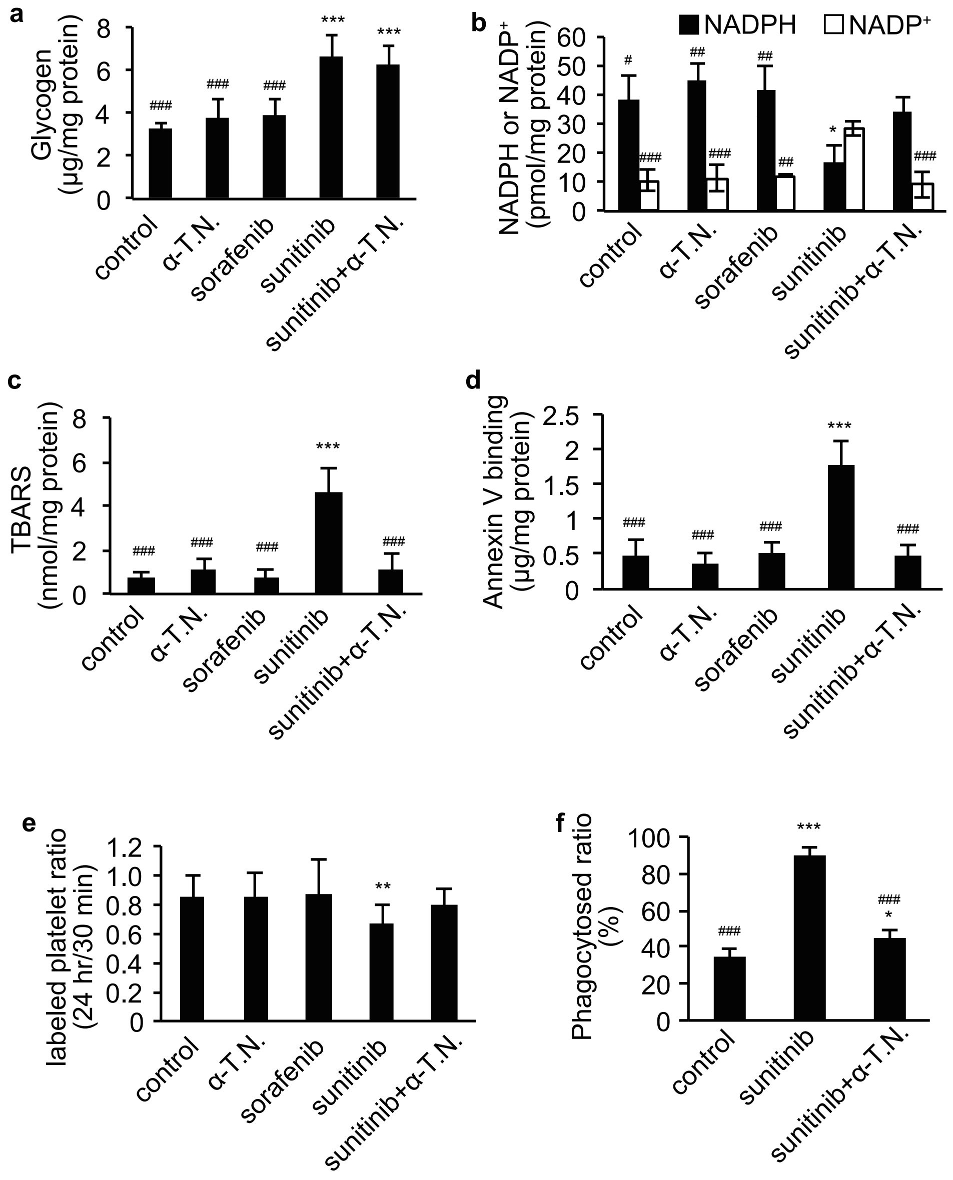

Fig. S7. Sunitinib-related oxidative stress leads to a decrease in platelet counts. Mice were fed sunitinib or sorafenib for 14 days after which the number of circulating platelets and the glycogen metabolite contents of these cells were analyzed. **a.** Platelet glycogen content was increased in sunitinib-treated mice. **b.** The ratio of NADPH to $NADP^+$ was decreased in the platelets of sunitinib-treated mice whereas the concomitant administration of α-tocopherol nicotinate (α-T.N.) maintained both ratios at the control levels. **c -d.** TBARS content (**c**.) and the binding of annexin V to the cell surface (**d**.) were increased in the platelets of sunitinib-treated mice whereas the concomitant administration of α-T.N. maintained the control levels of both. **e.** The half-life of circulating platelets was decreased in sunitinib-treated mice whereas the concomitant administration of α-T.N. maintained the half-life at the control level. **f.** Macrophage phagocytosis of platelets was enhanced in sunitinib-treated mice. All data are expressed as the mean ± SD (n = 6). * $P < 0.05$, ** $P < 0.01$, *** $P < 0.001$ v.s. Control; # $P < 0.05$, ## $P < 0.01$, ### $P < 0.001$ v.s. Sunitinib.

Figure S8

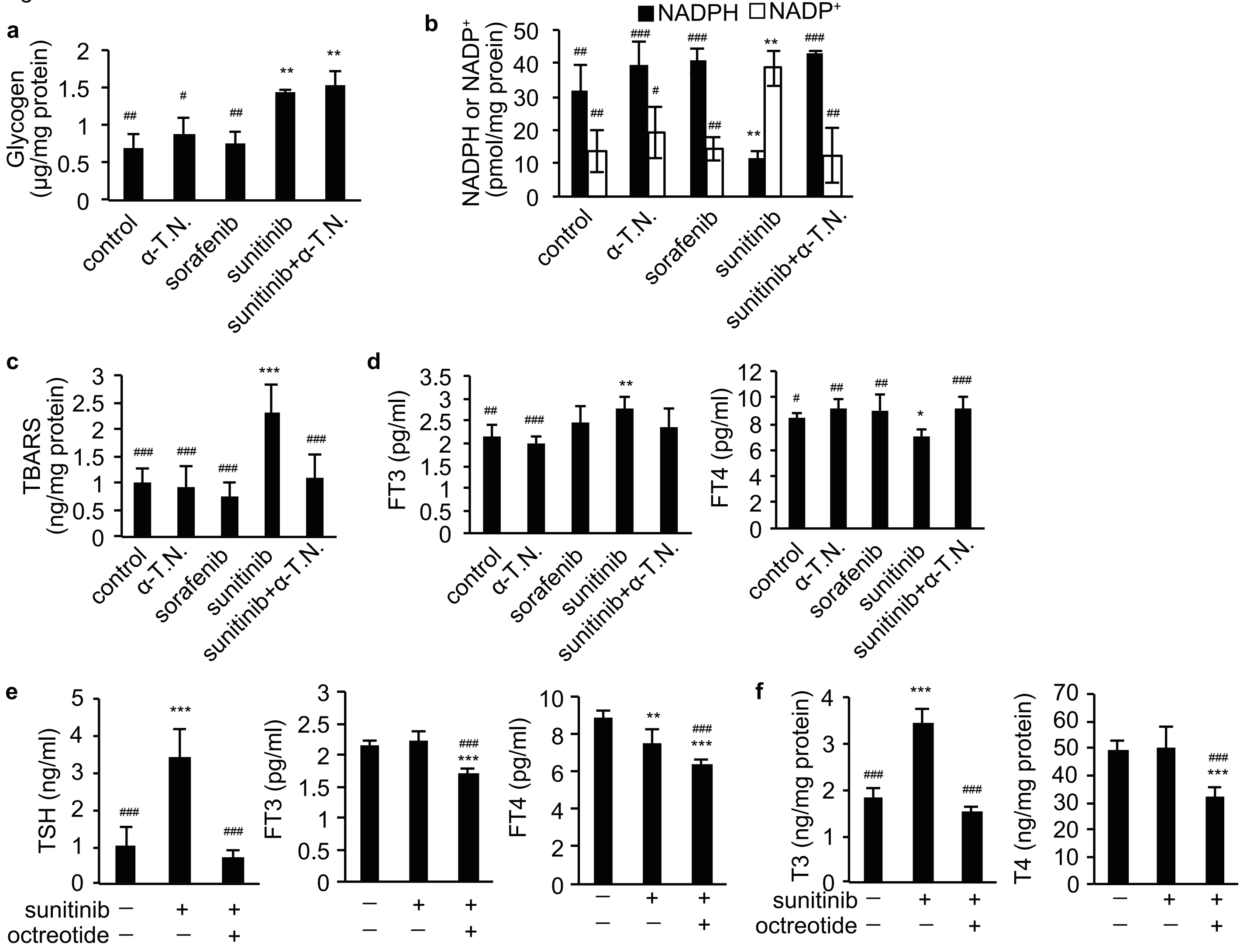

Fig. S8. Sunitinib-related oxidative stress is involved in thyroid dysfunction. Mice were fed sunitinib or sorafenib for 14 days after which serum biomarkers and thyroidal glycogen metabolite levels were analyzed. **a.** Thyroidal glycogen content was increased in sunitinib-treated mice. **b.** The ratio of NADPH to $NADP^+$ was decreased in the thyroid glands of sunitinib-treated mice whereas the concomitant administration of α-tocopherol nicotinate (α-T.N.) maintained both ratios at the control levels. **c.** Thyroidal TBARS levels were increased in sunitinib-treated mice whereas the concomitant administration ofα-T.N. resulted in the maintenance of the control level. **d.** The serum FT4 concentration was only slightly decreased in sunitinib-treated mice while the FT3 value remained unaffected. **e -f.** The sunitinib-mediated elevation in TSH levels was suppressed by the co-administration of octreotide, resulting in significant reductions in serum FT3 and FT4 levels (**e**.). Thyroid T3 and T4 levels (**f**.) were also significantly decreased. All data are expressed as the mean ± SD (n = 6). * $P < 0.05$, ** $P < 0.01$, *** $P < 0.001$ v.s. Control; # $P < 0.05$, ## $P < 0.01$, ### $P < 0.001$ v.s. Sunitinib.
